# Immune profiling and functional analysis of NK and T cells in ataxia telangiectasia

**DOI:** 10.3389/fimmu.2024.1377955

**Published:** 2024-08-06

**Authors:** Lea Graafen, Annekathrin Heinze, Nawid Albinger, Emilia Salzmann-Manrique, Franziska Ganß, Sabine Hünecke, Claudia Cappel, Sandra Wölke, Helena Donath, Jordis Trischler, Till-Martin Theilen, Christine Heller, Christoph Königs, Stephan Ehl, Peter Bader, Thomas Klingebiel, Jan-Henning Klusmann, Stefan Zielen, Ralf Schubert, Evelyn Ullrich

**Affiliations:** ^1^ Department of Pediatrics, Goethe University Frankfurt, Frankfurt am Main, Germany; ^2^ Department of Pediatrics, Experimental Immnology and Cell Therapy, Goethe University Frankfurt, Frankfurt am Main, Germany; ^3^ Frankfurt Cancer Institute, Goethe University, Frankfurt am Main, Germany; ^4^ Department of Pediatrics, Division of Pneumology, Allergology, Infectious Diseases and Gastroenterology, Goethe University Frankfurt, Frankfurt am Main, Germany; ^5^ Department of Pediatric Surgery and Urology, Goethe University Frankfurt, Frankfurt, Germany; ^6^ Institute for Immunodeficiency, Center for Chronic Immunodeficiency, Medical Center-University of Freiburg, Faculty of Medicine, University of Freiburg, Freiburg, Germany; ^7^ German Cancer Consortium (DKTK) Partner Site Frankfurt/Mainz, Frankfurt am Main, Germany

**Keywords:** ataxia telangiectasia, NK cells, T cells, immune characterization, phenotyping

## Abstract

Ataxia telangiectasia (AT) is a rare autosomal-recessive disorder characterized by profound neurodegeneration, combined immunodeficiency, and an increased risk for malignant diseases. Treatment options for AT are limited, and the long-term survival prognosis for patients remains grim, primarily due to the emergence of chronic respiratory pathologies, malignancies, and neurological complications. Understanding the dysregulation of the immune system in AT is fundamental for the development of novel treatment strategies. In this context, we performed a retrospective longitudinal immunemonitoring of lymphocyte subset distribution in a cohort of AT patients (*n* = 65). Furthermore, we performed FACS analyses of peripheral blood mononuclear cells from a subgroup of 12 AT patients to examine NK and T cells for the expression of activating and functional markers. We observed reduced levels of peripheral blood CD3^+^CD8^+^ cytotoxic T cells, CD3^+^CD4^+^ T helper cells, and CD19^+^ B cells, whereas the amount of CD3^−-^CD56^+^ NK cells and CD3^+^CD56^+^ NKT-like cells was similar compared with age-matched controls. Notably, there was no association between the age-dependent kinetic of T-, B-, or NK-cell counts and the occurrence of malignancy in AT patients. Additionally, our results indicate an altered NK- and T-cell response to cytokine stimulation in AT with increased levels of TRAIL, FasL, and CD16 expression in NK cells, as well as an elevated activation level of T cells in AT with notably higher expression levels of IFN-γ, CD107a, TRAIL, and FasL. Together, these findings imply function alterations in AT lymphocytes, specifically in T and NK cells, shedding light on potential pathways for innovative therapies.

## Highlights

- In a large cohort of AT patients (n = 65), we observed reduced levels of B cells, CD4^+^ cells, and CD8^+^ T cells and no difference in the amount of NK and NKT cells in peripheral blood compared with age-matched reference values.
- Longitudinal evaluation of immune cell counts in the same AT cohort did not predict the subsequent development of malignancies.
- Extended immune phenotyping indicates altered expression of death receptor ligands and CD16 in stimulated NK cells together with a higher activation level of T cells in AT, demonstrated in the increased expression of IFN-γ, CD107a, TRAIL, and FasL.

## Introduction

Ataxia telangiectasia (AT) is a rare autosomal recessive disorder, presenting with severe neurodegeneration, immunodeficiency, a considerably increased risk for malignant diseases, and a devastating prognosis (1). In the disease pathogenesis, a genetic defect in *ataxia-telangiectasia-modified*-gene (*ATM*-gene) on chromosome 11q22–23 leads to dysfunction of the ataxia-telangiectasia-modified protein (ATM-protein) (1), a kinase mainly involved in the repair of DNA-double-strand breaks (2, 3). Defects in the ATM-protein have impact on important immunological functions such as cell cycle control (4), meiotic recombination, and recombination in immunoglobulin genes, which are important for the rearrangement of the B- and T-cell receptor (BCR; TCR) genes and class switch of mature B cells (1, 5, 6).

There are two major distinct AT phenotypes differing in disease progress. While *classical AT* patients show a complete loss of ATM functionality and usually present with high morbidity, in *variant AT* patients, a certain functionality of the ATM protein can be observed, leading to a milder clinical course of disease and longer survival (7–9). In addition, patients with *classical AT* with IgA deficiency have significantly lower lymphocyte counts and subsets, which are accompanied by reduced survival, compared with AT patients without IgA deficiency (10, 11).

The two major causes of death in AT are malignant diseases and chronic pulmonary pathologies (1, 12, 13), which are both likely linked to the immunological dysfunctions (14, 15). In this regard, it is a key factor to understand the underlying mechanisms of immunodeficiency in AT. Up to now, our understanding indicates impairment in both the cellular and humoral immune system in affected patients. They often present with severe lymphopenia and reduced levels of IgG2, IgA, and IgE antibodies (16–19). Within the lymphocyte population, decreased numbers of T and B cells can be observed whereas an elevated number of NK cells have been suggested (16). Within the T-cell population, there is a lack of naïve (CD45RA^+^) CD4^+^ and CD8^+^ T cells, whereas numbers of T memory cells (CD45RO^+^) remain in the normal range (16, 20).

In the context of immunodeficiency in AT, recurrent infections mainly of the respiratory tract play a major role. In some cases, also granulomas can emerge, which are usually restricted to the skin (21). As a pathophysiological mechanism, a dysregulation of the immune system, involving among others macrophages, T cells, and NK cells, has been supposed and treatment with TNF inhibitors was found to be successful in some patients (21).

Since NK cells play a major role in the first line of defense against pathogens and are crucial for the recognition and elimination of malignant cells, we investigated their functionality in ATM deficiency. In parallel, T cells were studied, which are known for their elementary role in the adaptive immune response and were already reported with altered functions in AT (16). The aim was to perform a detailed cellular immune profiling of NK and T cells in AT to better understand the existing immunodeficiency.

Therefore, retrospective lymphocyte phenotyping data were collected from a total of 65 AT patients from the Department of Pediatrics at the University Hospital Frankfurt. The longitudinal course of T, B, NK, and NKT-like cells was compared with corresponding age-dependent reference values (22, 23), and the association between the immunological profile of AT patients and the occurrence of malignant diseases was studied.

For further functional FACS analysis, primary blood samples of 12 AT patients of the same cohort as well as primary blood samples of 17 healthy controls were collected and analyzed. We specifically investigated two different cytotoxicity pathways, the granzyme-perforin-dependent and the death receptor-dependent one, as well as CD107a and IFN- γ expression as markers of NK- and T-cell degranulation, cytotoxicity, and cytokine production (24). Important clinical data on this subgroup were collected retrospectively by reviewing patient records.

Overall, the identification of changes in NK- and T-cell phenotype and functionality in AT is fundamental, not only to understand the disease progress but also to possibly support developing novel treatment strategies such as supportive cell therapy concepts in the future.

## Materials and methods

The study design consists of a retrospective, longitudinal analysis of immune phenotype in AT patients as well as a cross-sectional analysis of more extensive NK- and T-cell phenotyping using functional markers.

### Study cohort

The study collected retrospective data from 65 AT patients of the Department of Pediatrics of the University Hospital Frankfurt. Diagnosis of AT was based on clinical and/or genetic findings. All patients with available flow cytometry data were included, except for patients with *variant AT* phenotype or participants of pharmacological clinical studies. Collected data comprise age; gender; the absolute numbers of T subsets, NK, and B cells; the development of malignant disease and its date; and the time of decease if applicable. Measurements of immune cell subsets were performed within the framework of patient monitoring and corresponds to its clinical requirements. Observation started as early as data of the individual patients was available and was limited to the end of the year 2022.

Additionally, blood samples from 12 of the 65 AT patients and 17 immunological healthy controls were collected in the cross-sectional study. Blood samples of AT patients were collected at one timepoint during routine check-up examinations between October 2018 and January 2020, whereas immunological healthy individuals presented for minor elective surgery or for consultation in the pediatric coagulation clinic during the same time period. The medical history of healthy controls revealed no signs of immune deficiency, allergic, autoimmune, or malignant diseases. Furthermore, clinical and if available also laboratory results did not show any inflammation and participants did not receive any medication affecting the immune system.

The study was accepted by the ethics committee of the University Hospital Frankfurt (#168/18), and either study subjects of legal age or custodians of underage patients agreed to participate.

### Isolation of PBMCs

Ethylenediaminetetraacetic acid- (EDTA-) anticoagulated blood was collected from all subjects and stored for up to 12 h at room temperature (RT). Isolation of PBMCs was conducted by centrifugation using cell separation solution (Biochrom, Merck Millipore, Burlington, MA, USA). Therefore, EDTA-blood was diluted with Dubecco’s phosphate-buffered saline (DPBS, Gibco™ Invitrogen/Thermo Fisher, Waltham, MA, USA), layered on the cell separation solution, and centrifuged for 20 min at 800*g* without break. The upper layer was transferred into another vessel, again diluted with DPBS, and centrifugated. The liquid was discarded, and the cell pellet was resuspended in DPBS. This washing step was executed twice before the cell pellet was taken up in 5 ml RPMI++ (Roswell Park Memorial Institute (RPMI, Gibco™ Invitrogen/Thermo Fisher) 1640 medium with 1% penicillin–streptomycin (Gibco™ Invitrogen/Thermo Fisher) and 10% fetal calf serum (FCS, Gibco™ Invitrogen/Thermo Fisher). The number of isolated PBMCs was determined using an Act Diff Cellcounter (Beckman Coulter, Brea, CA, USA). Subsequently, the cell suspension was again centrifugated and the cell pellet taken up in freezing solution (70% RMPI++, 10% dimethyl sulfoxide (DMSO, Sigma Aldrich, St. Louis, MO, USA), and 20% FCS) at concentrations of 1–10 × 10^6^ PBMCs/ml. The PMBCs were stored at −80°C for at least 24 h using a Mr. Frosty™ freezing container (Thermo Fisher Scientific, Waltham, MA, USA) and afterward transferred into liquid N_2_ for long-term storage.

### Flow cytometry-based surface phenotyping

Flow cytometry analyses were used to detect lymphocyte populations and a broad repertoire of functional markers on NK and T cells. Therefore, previously frozen PBMCs were thawed in a water bath at 37.6°C and then transferred into 40-ml preheated RPMI++. After centrifugation, the cell pellet was resuspended in 1 ml RPMI ++ and the cell count as well as life–death ratio were detected using a Neubauer counting chamber (Marienfeld, Lauda Königshofen, Germany).

Subsequently, depending on blood sample volume, 0.025 × 10^6^ and 0.2 × 10^6^ PBMCs were washed with 1 ml DPBS. The supernatant was discarded, and the cell pellet was first resuspended in FACS buffer [Cell Wash (Becton Dickinson, Franklin Lakes, NJ, USA) + 0.5% bovine serum albumin (BSA, Sigma Aldrich) + 0.01% NaN_3_ (Carl Roth GmbH, Karlsruhe, Germany)] and then incubated for 20 min with the following fluorochrome-conjugated antibodies: CD45-BV510 (clone HI30), CD3-BUV395 (clone SK7), CD56-BV421 (clone NCAM16.2), CD19-FITC (clone HIB19) (all from Becton Dickinson), CD14-BV711 (clone M5E2), and CD16-PE (clone 3G8) (all from BioLegend, San Diego, CA, USA). Afterward, the cells were washed and incubated for 5 min with 7-aminoactinomycin (7-AAD, Becton Dickinson) in FACS buffer.

Antibody-labeled cells were analyzed using a FACSCelesta™ instrument (Becton Dickinson). Data analysis was conducted using FlowJo^®^ (Becton Dickinson).

### Cell culturing for functional analyses

Based on the phenotyping results, NK cell count was adjusted to 0.1 × 10^6^ or 0.2 × 10^6^ NK cells/ml and cells remained either unstimulated (U) or received stimulation 1 (S1) or stimulation 2 (S2). Unstimulated cells were diluted 1:1 in RPMI++. For stimulated cells, additionally IL-2 (10^4^ U/ml) (Novartis, Basel, Switzerland) and IL-15 (0.01 µg/ml) (PeproTech Inc., Thermo Fisher) were added. If possible, technical duplicates or triplicates were evaluated. Subsequently, cells were incubated for 17 h–19 h at 37°C and 5% CO_2_. Then, an anti-CD107a-APC antibody (Clone H4A3, BioLegend) was added to specific conditions, followed by addition of monensin (GolgiStop™, Becton Dickinson, prediluted 1:10 with RPMI++) 1 h later to all wells. Stimulated cells received an additional boost with IL-12 (100 ng/ml) (PeproTech Inc., Thermo Fisher) and IL-18 (100 ng/ml) (MBL International Corp., JSR Micro, Leuven, Belgium) for S1 and with phorbol 12-myristat-13 acetate (PMA) (50 ng/ml) (Sigma Aldrich) and ionomycin (500 ng/ml) (PromoCell, Heidelberg, Germany) for S2. Cells were incubated at 37°C and 5% CO_2_ for 2 h.

### Intracellular flow cytometry analyses

After cultivation, cells were washed and resuspended in DPBS. Subsequently, the cells were incubated with 1 mg/ml immunglobulin G (IgG, Intratect^®^, Biotest Pharma GmbH, Dreieich, Germany) and with 0.05 µl Zombie Violet™ (BioLegend)/µl cell suspension for 15 min at RT. After washing, cells were incubated with anti-CD56-PE (clone NCAM16.2, Becton Dickinson), anti-CD16-BV511 (clone 3G8, Becton Dickinson), and anti-CD3-PerCP (clone UCHT1, BioLegend) antibodies and incubated for 20 min at 4°C. To specific conditions, anti-FasL-APC (clone NOK-1, Becton Dickinson) or anti-TRAIL-APC (clone RIK2, BioLegend) was added.

For intracellular staining, cells were fixated with 100 µl formaldehyde 37% (AppliChem GmbH, Darmstadt, Germany) (15 min, 4°C). After washing, cells were stained in saponin buffer (DPBS + 0.2% saponin + 1% BSA) with anti-IFN-γ-FITC (clone B27, Becton Dickinson) or with anti-Granzyme B (GrzB)-FITC (clone REA22, BioLegend) antibodies and incubated for 30 min at 4°C. Lastly, cells were washed in 1 ml Perm/Wash™ buffer (Becton Dickinson) (prediluted 1:10 with distilled water) and resuspended in Perm/Wash™ buffer to be analyzed using a FACSCanto™ 10C device (Becton Dickinson). The gating strategy for the analysis of FACS data is illustrated in **Supplementary Figure S2**.

### Statistical analysis

The joint model was used to track changes in the longitudinal trajectories of each lymphocyte subpopulation and their effect on the development of a malignancy. The longitudinal submodel was a B-spline linear mixed effect model. The Cox model fitted the time to develop a malignancy as an event taking into account death without previous cancer as a competing event and patients without any event as censored.

Univariate standardized mixed models were performed to characterize differences with age-reference models. For standardization, we took the lower limit of the 95% confidence interval of the reference values, and the standardized values were logarithmically transformed before running the mixed model to obtain an approximately Gaussian distribution for the residuals. Analyses were performed with the use of R-4.2 (R Foundation for Statistical Computing, Vienna, Austria). The nlme package version 3.1–162 and JM package version 1.5–2 were used. FACS data were analyzed using FlowJo^®^ version 8–10 (FlowJo LLC, Ashland, ORE, USA) and analyzed using unpaired t-tests and linear mixed models with GraphPad PRISM version 6–8 (GraphPad Software, Inc., San Diego, CA, USA) as well as R-4.2. A p-value of <0.05 was considered significant.

## Results

### Clinical characteristics of AT patients

For this study, 65 AT patients were included of whom 57% were men and 43% were women. The median survival since birth was 27 years. There were 14 patients that developed a malignancy resulting in an incidence of 27.4% (95% CI 11.6–43.2) and 50.65% (28.7–72.6) at 20 years and at 30 years, respectively (**Supplementary Figure S1**). The malignant disease consisted of leukemia (n = 4), non-Hodgkin-lymphoma (n = 6), Hodgkin-lymphoma (n = 3), and meningioma (n = 1). The median age of malignancy onset was 13 years (range 1.24–29). The estimated incidence at 20 and 30 years was 7.7% (0.0–18.1) and 19.7% (1.5–37.9). Six patients died without having developed any malignant disease. The cause of death in three of these cases was respiratory failure, either due to acute infectious pneumonia (n = 1) or on the basis on chronic restrictive lung disease (n = 2). In the other three patients, causes of death are unknown based on the available patient records.

In the subgroup cohort from 12 patients (**Table 1**), median age at time of blood collection was 3.5 years (range 1–26) and five of the patients were wheelchair bound (ID1, ID2, ID4, ID5, ID12) as a sign for advanced neurological impairment. Only AT1 developed a malignant disease up to the timepoint of measurement—a lymphoma. Three patients received Ig-replacement therapy due to hypogammaglobulinemia, deficiency of IgG subclasses, and/or a history of frequent infections. The same patients had a medication history of adalimumab due to the development of cutaneous granulomas (ID2, ID5, ID12), whereas only in one patient (ID5) it was still administered at the timepoint of measurement. Both therapies were administered in a subcutaneous way lastly. One more patient showed cutaneous granulomas but did not receive immunomodulating therapy for this indication (ID5).

**Table 1:** Characteristics of the AT subgroup of 12 patients.

**ID-Nr.**	**1**	**2**	**3**	**4**	**5**	**6**	**7**	**8**	**9**	**10**	**11**	**12**
**Sex**	m	m	f	m	f	f	m	m	m	m	m	f
**Age (years)**	26	19	3	12	20	2	1	2	3	4	2	21
**Malignant diseases**	#1	–	–	–	–	–	–	–	–	–	–	–
**Wheelchair dependence**	+	+	–	+	+	–	–	–	–	–	–	+
**Granuloma**	–	+	–	**+**	+	–	–	–	–	–	–	+
**Ig substitution**	–	+	–	–	+	–	–	–	–	–	–	+
**Adalimumab therapy**	–	+			+			–	–	–	–	+

Displayed are sex, age, and history of Ig-substitution or adalimumab therapy, cancer development, wheelchair dependency, and granuloma development at timepoint of analysis. The diagnosed malignant disease in ID1 was lymphoma (#1).

In the healthy control group, 13 participants were men and 4 were women. The median age was 8 years (range 1–19).

### Longitudinal immune cell analysis revealed reduced B and T cell but no difference in NK and NKT cell levels in the AT patient cohort

**Figure 1:**
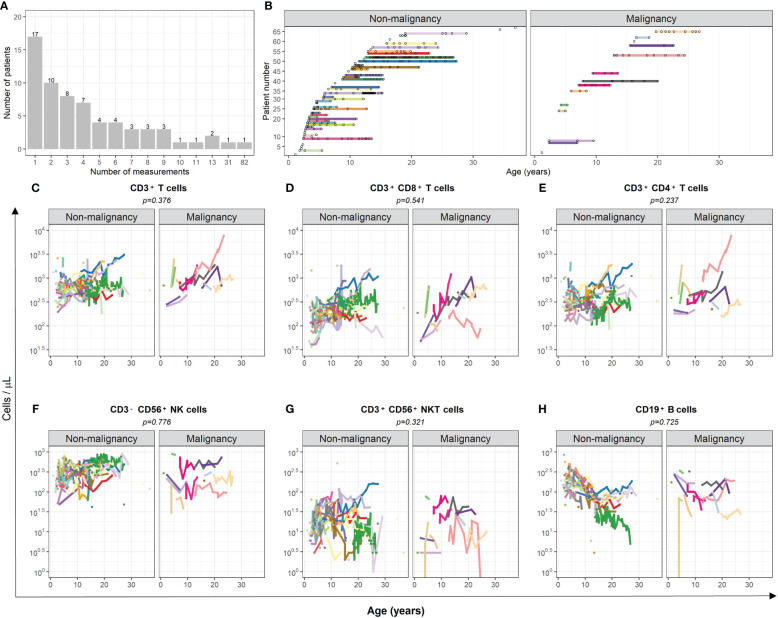
Comparison of cell numbers of different immune cell populations in peripheral blood of AT patients with and without subsequent development of malignancies. **(A)** The number of measurements of peripheral blood samples of each patient is displayed. **(B)** The time-point of each measurement of each individual patient is illustrated. **(C–H)** The total amount (cells/µl) of **(C)** CD3^+^ T cells, **(D)** cytotoxic CD3^+^CD8^+^ T cells, **(E)** CD3^+^CD4^+^ T helper cells, **(F)** CD3^-^CD56^+^ NK cells, **(G)** CD3^+^CD56^+^ NKT-like cells, and **(H)** CD19^+^ B cells are compared between AT patients with and without subsequent development of malignancies. Measurements after confirmation of cancer diagnosis were excluded. For statistical analysis, a mixed-effect linear model was utilized. Each model considers age and the respective cell population as covariate. Results were determined as significantly different for *p* < 0.005.

Longitudinal data of peripheral blood comprised 305 samples from patients who stayed cancer free and 60 of patients who developed a malignancy, but data correspond prior to the diagnosis of malignancy. The patients’ age at the first measurement was in median 5.92 (range 1.09–36.86) years. Number of measurements per patient ranged from 1 to 82 (**Figures 1A, B**).

There was no evidence of association between the kinetics of T, NK, and B cells and the occurrence of malignancy in AT patients considering death without malignancy as competing risk event (CD3^+^ T cells *p* = 0.376; CD3^+^CD8^+^ T cells *p* = 0.541; CD3^+^CD4^+^ T cells *p* = 0.237; CD3^-^CD56^+^ NK cells *p* = 0.776; CD3^+^CD56^+^ NKT-like cells *p* = 0.321; CD19^+^ B cells *p* = 0.725; **Figures 1C–H**). One patient (light pink line) showed outstanding high numbers of T cells, which might be related to the subsequently occurring T-acute lymphoblastic leukemia (T-ALL). To avoid possible bias, the numbers of T and helper T cells of this patient were not considered in the analysis.

**Figure 2:**
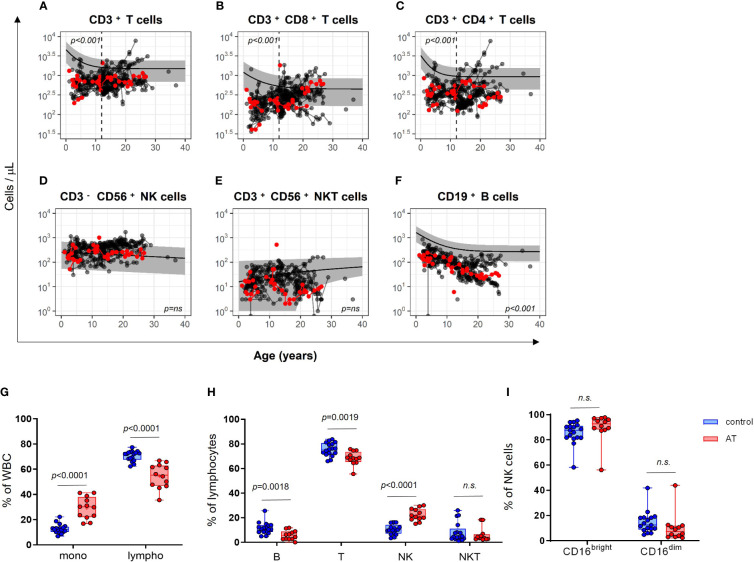
Comparison of cell numbers of different immune cell populations in peripheral blood of AT patients (*n* = 65) compared with healthy controls (the predicted mean reference values and its 95% confidence interval were obtained from reference model (22, 23). **(A–F)** The total amount (cells/µl) of **(A)** CD3^+^ T cells, **(B)** cytotoxic CD3^+^CD8^+^ T cells, **(C)** CD3^+^CD4^+^ T helper cells, **(D)** CD3^−^CD56^+^ NK cells, **(E)** CD3^+^CD56^+^ NKT-like cells and, **(F)** CD19^+^ B cells are compared longitudinally between AT patients and healthy controls. Red dots represent the 12 patients of the cohort, which was subsequently used for the analysis of functional markers. Measurements after confirmation of cancer diagnosis were excluded. For statistical analysis, linear mixed models were utilized. Each model considers age and the respective cell population as covariate. Results were determined as significantly different for *p* < 0.005. Phenotyping of white blood cells (WBC) of AT patients (AT) and healthy controls (C) analyzed with flow cytometry using isolated peripheral mononuclear cells (PBMC): **(G)** Monocytes and lymphocytes (% of WBC). **(H)** CD19^+^ B, CD3^+^ T, CD3^-^CD56^+^ NK, and CD3^+^CD56^+^ NKT-like cells (% of lymphocytes). **(I)** CD16^bright^ and CD16^dim^ subpopulations of NK cells (%). AT (red bars): *n* = 12, controls (blue bars): *n* = 17. Minimum, maximum, 25. and 75. percentiles are shown, as well as the median. For statistical analysis, unpaired t-test was used. Mono, monocytes; lympho, lymphocytes; B, B cells; T, T cells; NK, NK cells; NKT, NKT cells. n.s., not significant.

The longitudinal data were further compared with the corresponding age-matched reference values of healthy children and young adults (22, 23). As expected, the trajectory of absolute T cells and B cells was significantly lower in AT patients (CD3^+^ T cells *p* < 0.001; CD3^+^CD8^+^ T cells *p* < 0.001; CD3^+^CD4^+^ T cells *p* < 0.001; CD19^+^ B cells *p* < 0.001; **Figures 2A–C, F**). However, this applies to all T-cell populations only until the age of 12 years (vertical black dashed line), whereas it applies to the B-cell population for all ages. In contrast, the NK and NKT cell counts were similar in AT patients compared with reference values (**Figures 2A–F**).

The subcohort of 12 AT patients displayed similar differences in absolute cell counts of immune cell populations in peripheral blood compared with healthy controls as the whole AT cohort with 65 patients (**Figures 2A–F**, red dots). Regarding the white blood cell distribution (WBCs), the AT subgroup displayed a significantly reduced percentage of lymphocytes and a significantly increased percentage of monocytes (**Figure 2G**). Within the lymphocyte population, the portions of CD3^-^CD56^+^ NK cells were highly elevated, whereas the portions of CD19^+^ B cells and CD3^+^CD56^–^ T cells were both reduced compared with healthy controls (**Figure 2H**). NK cells displayed no differences in CD16^bright^ and CD16^dim^ subpopulations (**Figure 2I**).

### Phenotypic and functional AT immune cell assessment indicated increased expression of death receptor ligands in stimulated NK cells together with a higher activation level of T cells after CD3-independent stimulation

With the aim of further deciphering the possible functional impairment of innate or adaptive immunity, we performed a series of functional multicolor FACS analyses to assess the activating surface markers, degranulation capacity, and IFN-γ secretion capacity of NK and T cells.

**Figure 3:**
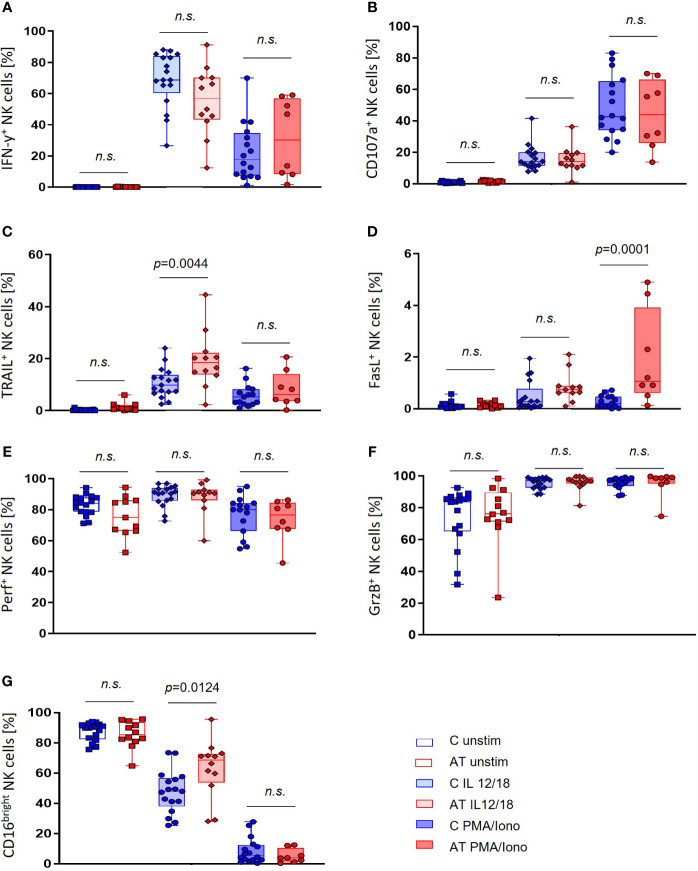
NK cell functional marker expression of AT patients (AT) and healthy controls (C) analyzed with flow cytometry using peripheral blood mononuclear cells (PBMC). Three different conditions were tested: unstimulated (unfilled bars), stimulation with IL-2/-12/-15/-18 (light filled bars), and stimulation with IL-2/-15/PMA/ionomycin (deep filled bars). **(A)** IFN-γ-expressing NK cells (%). **(B)** CD107a-expressing NK cells (%). **(C)** TRAIL-expressing NK cells (%). **(D)** FasL-expressing NK cells (%). **(E)** Perforin-expressing NK cells (%). **(F)** Granzyme B (GrzB)-expressing NK cells (%). **(G)** CD16^bright^ NK cells (%). Single values (black dots), minimum, maximum, 25. and 75. percentiles are shown, as well as the median. For statistical analysis, linear mixed models were used. AT (red bars): unstimulated *n* = 11–12, IL-12/-18 *n* = 11–12, PMA/ionomycin *n* = 8. Healthy controls (blue bars): unstimulated *n* = 17, IL-12/-18 *n* = 17, PMA/ionomycin *n* = 14–16. n.s., not significant.

Importantly, the portion of the cytotoxically active CD16^bright^ NK cell subset was elevated in AT NK cells after stimulation with IL-12/IL-18 (*p* = 0.0124 (**Figure 3G**), which mimics a physiological stimulation by factors of the monocytic and dendritic cell components. At the same time, the IFN-γ expression seemed to be slightly reduced in this population, whereas there was no statistically significant difference compared with the control group (**Figure 3A**). No differences between AT patients and healthy controls were further found in unstimulated or PMA/Iono-stimulated NK cells for both IFN-γ and CD16 expression.

Simultaneously, expression levels of TRAIL and FasL were significantly higher in AT patients compared with healthy controls after stimulation of NK cells (**Figures 3C, D**). In particular, the expression of TRAIL after stimulation with IL-12/18 (*p* = 0.0044) and the expression of FasL after stimulation with PMA/Iono were increased (*p* = 0.0001).

Within the NK-cell subset, no significant differences in the expression of CD107a, GrzB, and perforin could be observed between AT patients and healthy controls (**Figures 3B, E, F**). For GrzB and perforin, however, there were notably greater variances with outstanding minimum expression levels of these proteins in the AT group.

**Figure 4:**
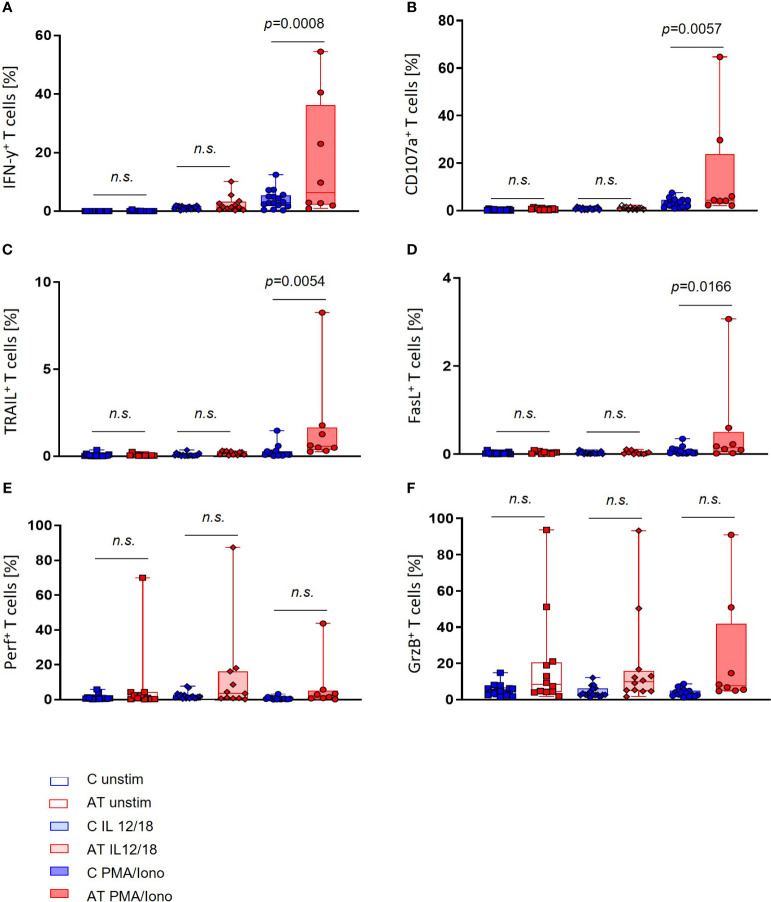
T-cell functional marker expression of AT patients (AT) and healthy controls (C) analyzed with flow cytometry using peripheral blood mononuclear cells (PBMC). Three different conditions were tested: unstimulated (unfilled bars), stimulation with IL-2/-12/-15/-18 (light filled bars), and stimulation with IL-2/-15/PMA/ionomycin (deep filled bars). **(A)** IFN-γ-expressing T cells (%). **(B)** CD107a-expressing T cells (%). **(C)** TRAIL-expressing T cells (%). **(D)** FasL-expressing T cells (%). **(E)** Perforin-expressing T cells (%). **(F)** Granzyme B (GrzB)-expressing T cells (%). Single values (black dots), minimum, maximum, 25. and 75. percentiles are shown, as well as the median. For statistical analysis, linear mixed models were used. AT (red bars): unstimulated *n* = 11–12, IL-12/-18 *n* = 11–12, PMA/ionomycin *n* = 8. Healthy controls (blue bars): unstimulated *n* = 17, IL-12/-18 *n* = 17, PMA/ionomycin *n* = 14–16. n.s., not significant.

Interestingly, regarding the T-cell functional markers, the portion of IFN-γ^+^, CD107a^+^, TRAIL^+^, and FasL^+^ cells after stimulation with PMA/Iono was significantly increased in the AT group compared with controls (IFN-γ: *p* = 0.0008, CD107a: *p* = 0.0057, TRAIL: *p* = 0.0054, FasL: *p* = 0.0166) (**Figures 4A–D**). Remarkably, great variances of values within the AT group could be observed, which were not visible in healthy controls.

Concerning the GrzB and perforin expression in T cells, no significant differences between AT patients and healthy controls could be detected, even if outliers with high portions of GrzB and perforin-expressing T cells were present for each stimulation (**Figures 4E, F**).

**Figure 5:**
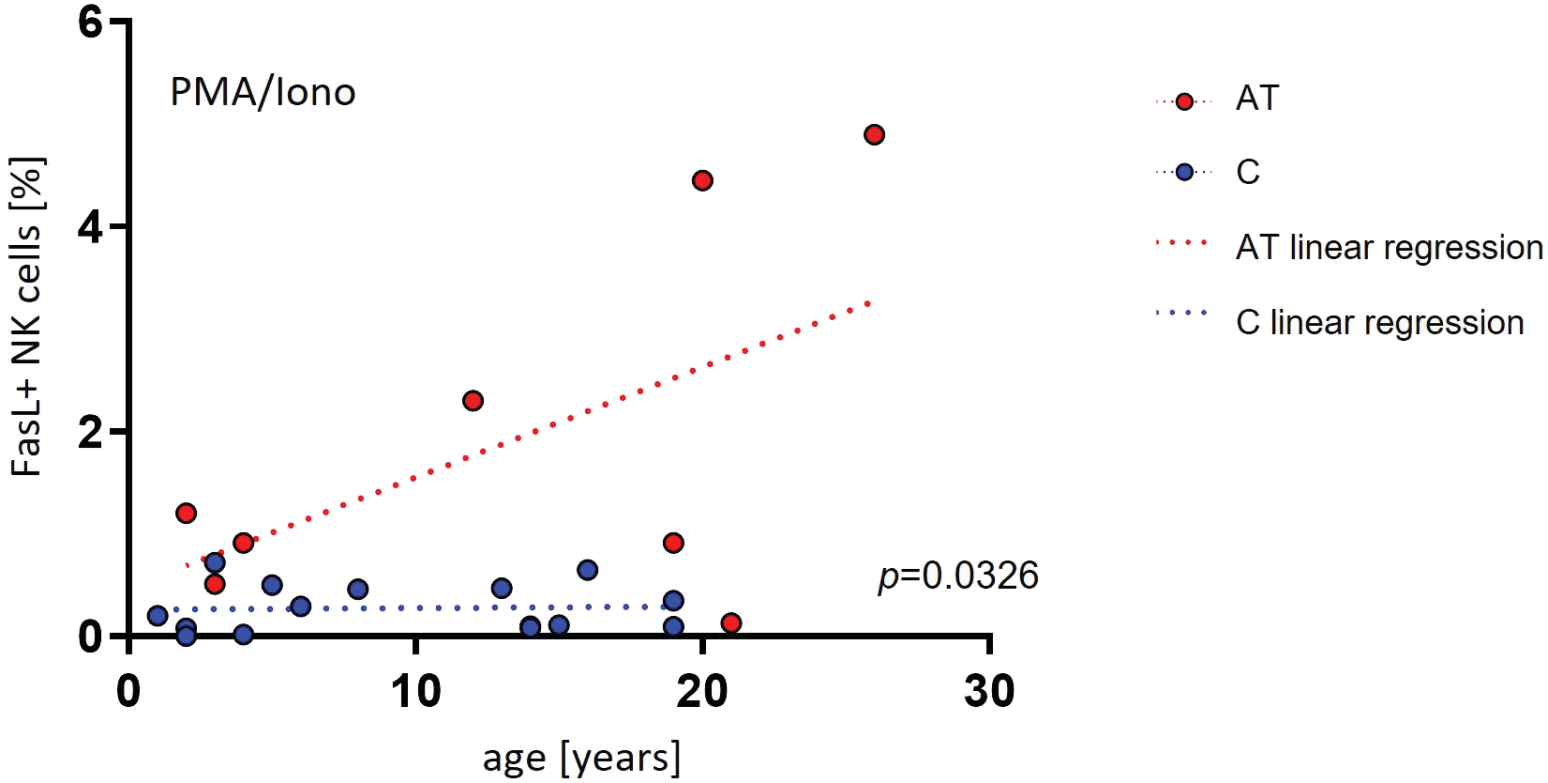
Age-dependent NK- and T-cell functional marker expression of AT patients (AT) and healthy controls (C) analyzed with flow cytometry using peripheral blood mononuclear cells (PBMC). Three different conditions were tested: unstimulated, stimulation with IL-2/-12/-15/-18, and stimulation with IL-2/15/PMA/ionomycin. Only statistically significant data are shown. FasL-expressing NK cells of AT patients and controls (%) after stimulation with PMA/ionomycin. The single values of AT patients (red dots) and healthy controls (blue dots) are shown, as well as the simple linear regression (AT = red line, controls = blue line). For statistical analysis linear mixed models were used. AT: *n* = 8. Healthy controls: *n* = 15.

Furthermore, we did not find any difference in the expression of functional markers of T cells in association with age between AT patients and healthy controls (data not shown). Concerning NK cells, however, there was a difference in the expression of FasL in association with age, with age being a positive predictor for FasL expression in AT NK cells (PMA/Iono p = 0.0326) (**Figure 5**).

## Discussion

In patients with AT, innovative therapy concepts are urgently needed to increase quality of life and life expectancy. Detailed knowledge about the functionality of the immune system in AT patients could contribute to the establishment of novel therapies, including cellular therapy approaches. Recently, we could demonstrate that stem cell transplantation as an individual preemptive treatment strategy is a new treatment approach from which some AT patients might benefit (25). We therefore conducted a phenotypical and functional immune cell profiling in AT patients.

Currently, we know that immunological dysfunction in AT is present in both the humoral and cellular parts of the immune system. On the cellular level, there is a reduction of B cells and of mostly naïve (CD45RA) CD4^+^ and CD8^+^ T cells (16). Yet, not only lymphopenia itself causes dysfunction of cellular immunity, but also an impaired stimulation of CD45RO T cells via the T cell receptor (TCR), which leads to decreased proliferation as well as IL-2 and IFN-γ production compared with healthy controls (16).

For NK cells, it is suggested that peripheral cell numbers are elevated in AT, which might be a compensatory mechanism to make up for the reduced number of T cells (16). However, also a reduced expression of NKG2D and perforin in NK cells of AT patients has recently been described, which went along with reduced NKG2D-mediated cytotoxicity (26). This indicates that alterations on a functional level could also play a role in AT NK cells.

Our analyses match these previous observations as we could also detect differences in lymphocyte subpopulations of AT patients and healthy individuals. Longitudinal data showed a significantly reduced number of both CD4^+^ and CD8^+^ T cells in AT patients up to the age of 12 years, as well as B cells throughout all ages compared with age-matched reference values. At the same time, however, the number of NK cells in our AT cohort was not statistically different from the reference values, so that no changes in absolute NK cell counts can be stated at this point. The fact that B and NK cells do not show age-dependent differences unlike T cells might be due to differences in cell generation and differentiation. The severe reduction in B-cell counts fits well to the observed hypogammaglobulinemia in AT patients.

Although hematological malignancies are one of the major causes of death, there was no association in the kinetics of lymphocyte subpopulations and the incidence of malignant disease in AT patients. We would therefore not suspect lymphocyte phenotype and its course to predict the evolution of malignancy in AT.

Furthermore, functional flow cytometry analyses revealed a shift in the distribution of NK-cell subpopulations toward cytotoxically more active CD16^bright^ NK cells after stimulation with IL-12/IL-18, whereas the immunoregulatory CD16^dim^ NK cell fraction seemed to be reduced. At the same time, the fractions of NK cells expressing the death receptor ligands TRAIL and FasL significantly differed. TRAIL expression in AT was particularly increased after stimulation with IL-12 and IL-18, and the expression of FasL was elevated after stimulation with PMA and Ionomycin. Since the death receptor-dependent cytotoxicity is involved in serial killing of pathogenic cells (27), the increased expression of these molecules in NK cells could indicate an activated NK-cell state in AT due to chronic recurrent infections.

Additionally, death receptors are involved in activation-induced cell death to maintain T-cell homeostasis (28, 29). As reconversion of CD45RO cells to CD45RA cells has been described, increased activation-induced cell death could lead to decreased reconversion to CD45RA cells. It could thereby contribute to the atypical distribution of naïve and activated T cells in AT lymphocytes (30). In this context, FasL expression on NK cells could be upregulated and induce apoptosis of activated T cells. Interestingly, the FasL expression of NK cells was highest following PMA and ionomycin stimulation, which also stimulates T cells the most. Whether the observed association between FasL expression and age in AT NK cells is thereby linked to the different kinetics in lymphocyte phenotype in AT, especially in the T-cell compartment, cannot be answered to this point and needs further evaluation.

Regarding the upregulation of CD107a in stimulated AT NK cells, however, as well as the expression of the apoptosis inducing molecules GrzB and perforin, no significant differences could be detected. We would therefore not assume function alterations of the granula-dependent killing machinery to be present in the context of AT disease, even though the killing potential against tumor cells or the alteration of downstream elements should be studied to further elaborate on this question.

Regarding T cells, our flow cytometry analyses revealed a significantly higher expression of IFN-γ, CD107a, TRAIL, and FasL after stimulation with PMA and ionomycin in AT patients compared with healthy controls, whereas no significant differences in the fractions of GrzB- and perforin-expressing cells could be observed. It is however noticeable that for all markers, including GrzB and perforin, individual patients with outstanding high portions of expressing T cells exist, so that pronounced interindividual differences in ATM deficiency can be assumed. The extent of functional marker expression throughout age did not differ significantly between the AT and the control group.

Together, this might suggest a generally higher activation level of T cells in AT, which fits to the higher portion of CD45RO cells observed in AT. Our results are also in accordance with Schubert et al., who similarly described a higher percentage of IFN-γ^+^ T cells, and with Pashankar et al., who found higher amounts of IFN-γ mRNA copies per cell in AT. At the same time, however, Schubert et al. observed an absolute decreased number of IFN-γ molecules per cell, which led to the hypothesis that T-cell activation might be on the contrary dysfunctional in AT.

Finally, it needs to be considered that an increase in the expression levels of the studied functional markers does not for itself mean an increase in functionality. A higher expression might also result from an underlying dysfunction together with compensatory mechanisms.

Furthermore, the longitudinal analysis of the AT cellular phenotype was conducted in a retrospective way and was therefore dependent on preexisting patient data. For several patients, therefore, only one measurement was available so that changes in immune phenotype over time cannot be evaluated.

Taking these limitations into account, the data presented clearly show differences in the lymphocyte phenotype of NK and T cells in AT. To evaluate how these differences translate into altered NK- and T-cell functionality, further studies are warranted.

Future studies should therefore address both the cytotoxic and regulatory potential of ATM deficient lymphocytes, as well as the phenotypic and genotypic differences in AT to meet demands of pronounced interindividual heterogeneity. A deeper comprehension of the underlying immunological dysfunctions in AT could be of importance for the development of immunotherapeutic concepts. In our opinion, novel personalized treatment strategies might be able to address different secondary diseases caused by the ATM deficiency such as the development of malignancies.

## Data availability statement

The raw data supporting the conclusions of this article will be made available by the authors, without undue reservation.

## Ethics statement

The studies involving humans were approved by Ethics Committee of the University Hospital Frankfurt. The studies were conducted in accordance with the local legislation and institutional requirements. Written informed consent for participation in this study was provided by the participants’ legal guardians/next of kin.

## Author contributions

LG: Formal analysis, Visualization, Writing – original draft, Writing – review & editing, Investigation. AH: Conceptualization, Investigation, Supervision, Writing – original draft, Writing – review & editing. NA: Supervision, Visualization, Writing – original draft, Writing – review & editing. ES-M: Data curation, Formal analysis, Methodology, Visualization, Writing – original draft, Writing – review & editing. FG: Investigation, Supervision, Writing – review & editing. SH: Resources, Writing – review & editing. CC: Resources, Writing – review & editing. SW: Resources, Writing – review & editing. HD: Resources, Writing – review & editing. JT: Resources, Writing – review & editing. T-MT: Resources, Writing – review & editing. CH: Resources, Writing – review & editing. CK: Resources, Writing – review & editing. SE: Writing – review & editing. PB: Writing – review & editing. TK: Writing – review & editing. J-HK: Writing – review & editing. SZ: Conceptualization, Resources, Writing – review & editing. RS: Conceptualization, Writing – review & editing. EU: Conceptualization, Funding acquisition, Project administration, Resources, Supervision, Writing – original draft, Writing – review & editing.

## Supplementary material

Supplementary Figure S1 Kaplan-Meier curves of **(A)** overall survival of the 65 AT patients since birth. OS estimate (black line) and 95% confidence intervals (shaded area) are illustrated. Median survival of the cohort was 27 years, six patients died without any malignancy. **(B)** cumulative incidence (CI) of malignancy and death without having presented a cancer. To estimate the CI of malignancy death before developing cancer was considered as competing event. The shaded area shows the respective 95% confidence intervals.

Supplementary Figure S2 Exemplary Flow Cytometry gating strategy to discriminate NK and T cells and to identify the expression of functional markers such as CD107a and IFN-γ.
