# Supplementary figures and images for: Immune profiling and functional analysis of NK and T cells in ataxia telangiectasia
Source: Front Immunol. 2024 Aug 6;15:1377955. doi: 10.3389/fimmu.2024.1377955 (PMC11333214; doi:10.3389/fimmu.2024.1377955)

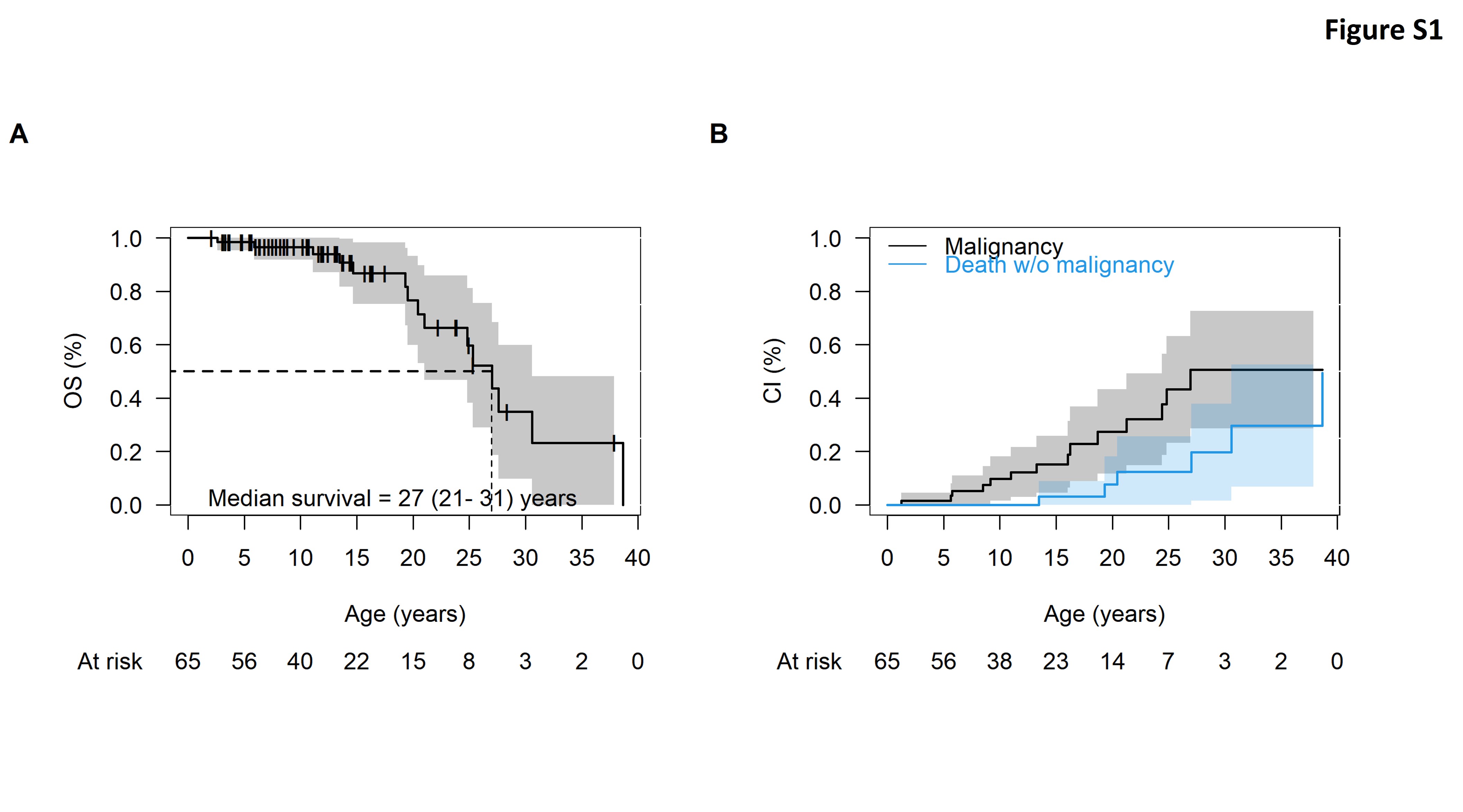

Supplement: Supplementary Figure S1 — Kaplan-Meier curves of (A) overall survival of the 65 AT patients since birth. OS estimate (black line) and 95% confidence intervals (shaded area) are illustrated. Median survival of the cohort was 27 years, six patients died without any malignancy. (B) cumulative incidence (CI) of malignancy and death without having presented a cancer. To estimate the CI of malignancy death before developing cancer was considered as competing event. The shaded area shows the respective 95% confidence intervals. [file Image_1.jpeg]

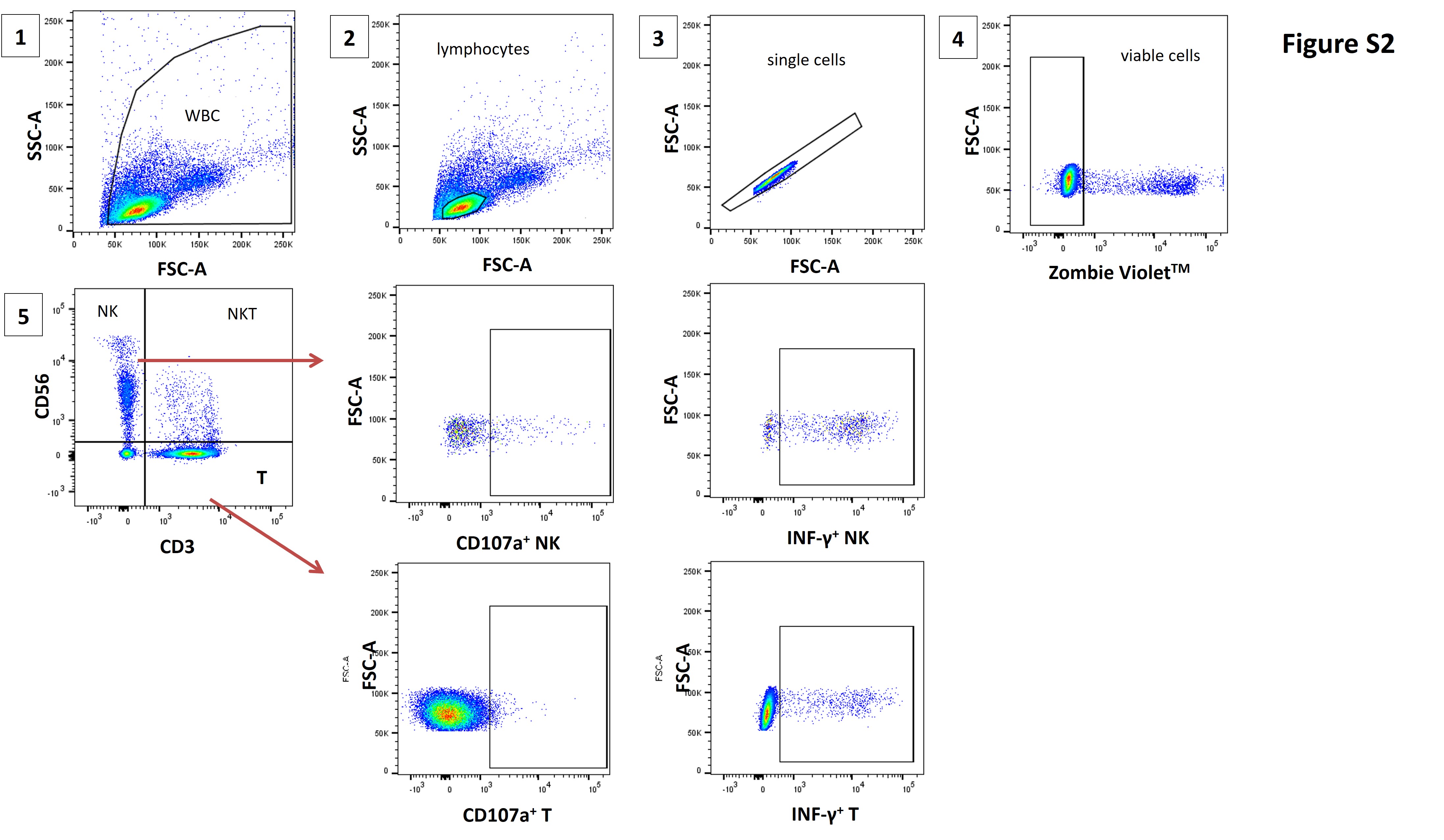

Supplement: Supplementary Figure S2 — Exemplary Flow Cytometry gating strategy to discriminate NK and T cells and to identify the expression of functional markers such as CD107a and IFN-γ. [file Image_2.jpeg]
